# Renal Vein Thrombosis: A Narrative Review

**DOI:** 10.3390/diagnostics16121805

**Published:** 2026-06-11

**Authors:** Nicoletta Riva, Alexander Gatt, Maria Angela Gauci, Lara Roberts, Jecko Thachil, Christian Borg-Xuereb

**Affiliations:** 1 Department of Pathology, Faculty of Medicine and Surgery, University of Malta, MSD 2080 Msida, Malta; alexander.gatt@um.edu.mt; 2 Division of Nephrology, Department of Medicine, Mater Dei Hospital, MSD 2090 Msida, Malta; maria-angela.gauci.1@gov.mt; 3 King’s Thrombosis Centre, Department of Haematological Medicine, King’s College Hospital NHS Foundation Trust, London SE5 9RS, UK; lara.roberts@nhs.net; 4 Department of Haematology, Manchester University Hospital, Manchester M13 9WL, UK; jecko.thachil@mft.nhs.uk; 5 Department of Gerontology and Dementia Studies, Faculty for Social Wellbeing, University of Malta, MSD 2080 Msida, Malta; christian.borg-xuereb@um.edu.mt

**Keywords:** venous thrombosis, anticoagulants, kidney, renal vein

## Abstract

Renal venous thrombosis (RVT) is a location of unusual-site venous thromboembolism. RVT occurs more commonly in males, and shows a bimodal age distribution, with a neonatal and adult peak. Abdominal malignancies and nephrotic syndrome are prominent risk factors in adults, whereas hypotension, birth asphyxia, sepsis, umbilical venous catheters and prematurity are the predominant causes in children. The most common symptoms of RVT include abdominal pain and macroscopic haematuria. A palpable abdominal mass is often observed in neonates, while antenatal RVT may present with signs of foetal distress. Bilateral RVT can lead to acute renal failure. Anticoagulation is the cornerstone of treatment, traditionally with unfractionated heparin, low molecular weight heparin and vitamin K antagonists, although recent evidence is emerging on the use of the direct oral anticoagulants in selected RVT patients. Endovascular procedures (e.g., local thrombolysis or mechanical thrombectomy) are usually reserved for more severe cases, such as bilateral acute RVT causing kidney dysfunction. Outcome data show variability in mortality rates, with some adult cohorts reporting high mortality linked to underlying malignancies and other comorbidities. In paediatric cohorts, mortality is low, but RVT can lead to long-term complications, including kidney atrophy, kidney dysfunction and hypertension. This narrative review aims to synthesise the current evidence on RVT, with a particular focus on anticoagulant prophylaxis and treatment, and clinical outcomes in adult and paediatric populations.

## 1. Introduction

**Figure 1:**
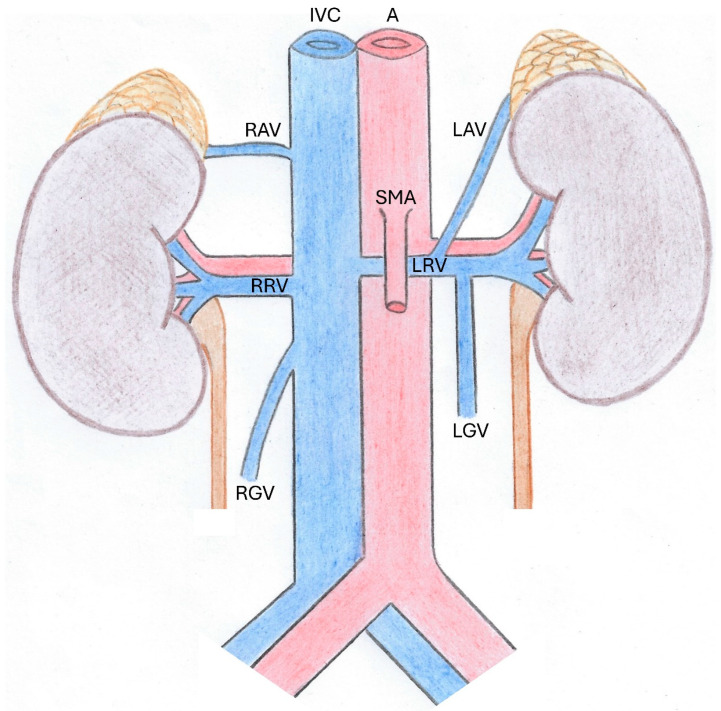
Course of the renal veins in the abdomen. Legend: A = aorta, IVC = inferior vena cava, LAV = left adrenal vein, LGV = left gonadal vein, LRV = left renal vein, RAV = right adrenal vein, RGV = right gonadal vein, RRV = right renal vein, SMA = superior mesenteric artery (Arteries are shown in red; veins are shown in blue).

Renal vein thrombosis (RVT) is a type of unusual-site venous thrombosis characterised by the development of a thrombus within the renal veins. The renal veins are formed by the convergence of multiple intrarenal branches (interlobular, arcuate, and interlobar veins) draining each kidney. They pass anterior to the corresponding renal arteries and drain into the inferior vena cava (IVC) at an approximately 90° angle (Figure 1). The left renal vein is longer than the right, passes between the aorta posteriorly and the superior mesenteric artery anteriorly, and along its course receives more tributaries, including the left gonadal vein and the left adrenal vein [1]. Since the thrombotic process in RVT usually begins in the small intrarenal veins and extends towards the main renal vein, some authors have suggested that the terminology “renal venous thrombosis” may be more accurate than “renal vein thrombosis” [2,3,4]; however, “renal vein thrombosis” is more commonly used in contemporary medical literature.

Despite the first description of RVT dating to the early 1840s, contemporary evidence remains fragmented, due to the scarcity of prospective studies, limited evidence guiding anticoagulation strategies, and lack of standardised management in particular clinical settings, such as paediatric patients and nephrotic syndrome (NS). This narrative review aims to summarise the latest evidence on RVT. To identify the available literature, we searched MEDLINE through PubMed up to 8th February 2026, using the following keywords: (“renal vein thrombosis”) OR (“renal thrombosis”) OR (“renal venous thrombosis”) OR (“renal vein” AND “thrombosis”).

## 2. Epidemiology

RVT is one of the least common manifestations of VTE. It represents 0.13–0.30% of all VTE cases, 3.34% of VTE at unusual sites, and 8.85% of abdominal vein thrombosis [5,6]. In neonates, RVT is more common, accounting for 12.5–15.5% of all VTE cases [7,8]. Nonetheless, neonatal RVT is considered a rare disorder and included in the Orphanet database (ORPHAcode: 664912). RVT may be underdiagnosed, due to frequent asymptomatic cases or nonspecific symptoms.

The incidence rate of RVT requiring hospitalisation was ≤0.1 per 100,000 person-years in a nationwide Swedish population-based register [5]. The incidence of symptomatic neonatal RVT was 2.2 per 100,000 among live births overall, and 13 per 100,000 among preterm births in Germany [9]. The annual incidence of neonatal RVT in Canada remained stable over time (between 1992 and 2016) at ~2.6 cases per 100,000 live births (range 1.6–3.4) [4].

There are no prevalence data in the general population, but it is well recognised that RVT is more frequent in certain patient groups. For instance, the prevalence of RVT was 22% in a Chinese cohort of patients with NS screened with computed tomography (CT) venography [10].

In general, RVT is more frequent in males, with a male-to-female ratio ranging from 1.1–1.5:1 [5,11,12,13,14,15] to 2.5–2.9:1 [2,10,16]. There are only very few studies showing a predominance of female sex with a ratio of 1:1.2–1.4 [17,18,19].

RVT shows a bimodal age pattern, with distinct neonatal and adult peaks. Paediatric cases are mainly diagnosed in the first weeks of life, with reported median ages of ~1–3 days [2,4,9,12,20], although they can sometimes be detected antenatally [2,7]. The age of presentation in adults varies based on the underlying pathogenesis: the average age was ~37–47 years in cohorts composed mainly of patients with NS [13,17,19,21], and ~55–64 years in cohorts composed mainly of patients with malignancy [6,18,22]. Furthermore, the median age of patients with RVT was lower than those with deep vein thrombosis (DVT) or pulmonary embolism (PE) (39 years vs. 69.5 years vs. 72.5 years, respectively) [17].

## 3. Pathophysiology and Risk Factors

Studies have reported bilateral RVT in ~10–40% of cases [16,18,20,23]. Amongst unilateral cases, the left renal vein is affected ~1.4–2.8 times more frequently than the right [2,12,16,18,21]. This can be due to its longer course, greater number of tributary veins, and the anatomical position in the passage between the abdominal aorta and the superior mesenteric artery, all factors which can predispose to slow blood flow (venous stasis) [24].

Unprovoked RVT is rare in paediatric cohorts and accounts for a minority of adult cases [14,22,25]. Indeed, one study reported that only 12% of RVT cases are idiopathic, compared to 23% of lower limb DVT [22], confirming that RVT most often arises from a combination of risk factors, consistent with a multifactorial pathogenesis.

The most common risk factors for RVT in adults are abdominal malignancies (mainly renal cell carcinoma, other genitourinary malignancies, and metastatic lung cancer [18,19]) and NS. Cancer predisposes to bland thrombosis of the renal vein by affecting all the three elements of the Virchow’s triad: venous stasis (e.g., extrinsic renal vein compression by a primary tumour, metastasis, or adjacent lymphadenopathy); endothelial injury (e.g., vascular wall damage associated with local tumour invasion); and hypercoagulability (e.g., release of procoagulant microparticles and cytokines) [26]. These mechanisms should be distinguished from direct tumour extension into the renal vein, which results in tumour thrombus rather than bland RVT. In an unselected cohort of adult patients with RVT, malignancy was present in 49% of cases, and patients with cancer-associated RVT were significantly older than non-cancer-associated RVT (mean age 63 vs. 37 years, *p* = 0.001) [14]. Furthermore, the prevalence of cancer was ~3 times higher in patients with RVT compared to patients with lower limb DVT (66.2% vs. 21.6%, *p* < 0.001) [22].

NS can also affect all three elements of the Virchow’s triad: venous stasis (e.g., intravascular volume depletion due to hypoalbuminemia); endothelial injury (e.g., inflammatory endothelial activation); and hypercoagulability at both systemic (e.g., increased hepatic synthesis of procoagulant factors and enhanced platelet activation, combined with urinary loss of natural anticoagulants, particularly antithrombin, but also protein C and protein S, and impaired fibrinolysis) and local levels (e.g., activation of the glomerular haemostatic system with intraglomerular thrombin formation) [27,28]. In a cohort of 7473 Japanese adult patients hospitalised with NS, 221 (3.0%) developed VTE, and RVT accounted for 5.0% of these cases [17]. In a study involving 1995 Chinese children with primary NS, 27 (1.4%) developed venous or arterial thrombosis, and RVT was the most common location of thrombosis, accounting for 33% of cases [29]. Minimal change disease and membranous nephropathy (MN) were the two most common histopathological subtypes of NS [29].

In neonates, RVT can be due to severe dehydration with hypotension, birth asphyxia, infection/sepsis, umbilical venous catheters, prematurity and maternal risk factors (such as maternal diabetes mellitus or hypertension) [30]. In the first days after birth, there is a delicate balance in coagulation status due to the peculiar neonatal haemostatic system, characterised by reduced levels of natural anticoagulant and fibrinolytic enzymes [25]. In a cohort of 2463 neonatal admissions in Thailand, 28 (1.1%) had arterial or venous thromboembolism, and RVT accounted for 12.5% of all VTE [8]. RVT can also occur antenatally in utero, causing foetal distress and potentially contributing to premature birth, which, according to some authors, may represent consequences of RVT, rather than risk factors for RVT [7].

RVT is a potential complication of kidney transplantation, occurring in ~1–3% of cases, generally in the first week after surgery. It is one of the most common causes of early graft loss, often necessitating allograft nephrectomy if the graft is non-viable [31,32,33]. Transplant RVT can result from a combination of donor- or recipient-related risk factors. Mechanical causes are among the most common, particularly compression or kinking of the renal vein from a peri-transplant fluid collection or stenosis of the venous anastomosis [34]. Allograft thrombosis may be more common with right donor kidneys due to the shorter right renal vein, which can pose technical challenges and prolong anastomosis time [35]. Surgical techniques can be employed to lengthen the right renal vein, using the donor’s IVC in deceased donor transplants or alternative vein grafting techniques in living donation [36]. The risk of thrombosis is also increased in paediatric transplantation due to smaller native vessel calibre [37]. Additional recipient-related risk factors include the presence of hypotension, acute rejection and the acute post-operative hypercoagulable state [34].

RVT can be a manifestation of inherited or acquired thrombophilia. Case–control studies assessing the role of hereditary thrombophilia reported high prevalence of Factor V Leiden and prothrombin G20210A mutations (37.3% and 8.5%, respectively) [11] and deficit of the natural anticoagulants protein C and antithrombin (5.0–6.5% and 3.2–5.0%, respectively) [11,38] in neonates with RVT, although the comparisons with healthy neonates were not always statistically significant. Renal vascular involvement has been reported in 3–10% of patients with antiphospholipid syndrome (APS), with a spectrum of diseases including renal artery, RVT, and thrombotic microangiopathy of the small intrarenal vessels [39,40]. The development of NS in APS should prompt the physician to investigate for RVT or a secondary cause of APS, such as systemic lupus erythematosus [39].

RVT has been reported in patients with autoimmune disorders, such as systemic lupus erythematosus [41], inflammatory bowel disease [42], Behçet disease [43,44], vasculitis and autoimmune haemolytic anaemia [45]. RVT may also occur following surgery or blunt abdominal trauma [46]. Acute pancreatitis can lead to extra-splanchnic abdominal venous thrombosis, such as RVT [47]. Other risk factors for RVT include infections, particularly urinary tract infections [48], COVID-19 [49,50] and vaccine-induced thrombotic thrombocytopaenia (VITT) [51]; paroxysmal nocturnal haemoglobinuria [52]; the use of combined hormonal contraception or other hormonal therapy (e.g., testosterone) [53,54,55]; and the puerperium, especially following a caesarean section [56]. Finally, RVT of the left renal vein has also been described as a complication of the ‘nutcracker’ syndrome (also known as left renal vein entrapment syndrome), a rare disease characterised by compression of the left renal vein at the passage between the aorta and the superior mesenteric artery [57].

## 4. Clinical Features

RVT can have acute or chronic onset, depending on the rapidity of venous occlusion and the formation of venous collateral circulation. In a cohort study of 39 patients with RVT in Israel, 64.1% had acute presentation (duration of symptoms <3 weeks), 20.5% chronic presentation (duration of symptoms >3 weeks) and 15.4% were asymptomatic [14]. In another cohort study of 87 adult patients with RVT in Thailand, 24.1% had acute onset, 10.3% chronic onset and 65.5% were asymptomatic [18]. Asymptomatic RVT are usually detected incidentally at radiological imaging performed during cancer staging [18].

Neonatal RVT was classically described by the diagnostic triad of palpable abdominal mass, macroscopic haematuria and thrombocytopenia. While this triad is present only in a minority of patients (13–22% of cases), the individual symptoms are commonly described in paediatric patients: palpable mass (39–62%), haematuria (35–48%), and thrombocytopenia (35–80%) [2,15]. The mechanism underlying thrombocytopenia in RVT is not clearly elucidated; however, thrombosis itself is reported as a cause of thrombocytopenia in neonates, likely due to platelet consumption [58].

RVT can also occur antenatally and lead to foetal distress [25]. Antenatal onset is suspected when RVT is diagnosed immediately after birth, especially if calcifications are detected at imaging [2].

In a cohort of 218 mainly adult patients with RVT, the most common presenting symptoms were abdominal pain (73%) and macroscopic haematuria (36%) [22]. Abdominal pain is typically localised to the affected flank and may be associated with kidney tenderness, while a palpable kidney is more commonly reported in paediatric cohorts. These findings reflect enlargement of the involved kidney due to venous congestion and capsule distension secondary to venous obstruction [21,59]. Approximately 40% of the patients may present with nonspecific symptoms (such as nausea, anorexia, or fever), while presentation with an acute abdomen has been rarely reported (4%) [22]. In the elderly, RVT tends to have a more chronic and insidious onset [14,21]. While kidney function is generally preserved in unilateral RVT, bilateral RVT can lead to severe acute kidney injury (AKI), which may be life-threatening in the short-term and may predispose to hypertension in the long-term [7].

RVT after kidney transplant usually occurs within 48 h of surgery. When it occurs intra-operatively, the allograft typically appears swollen and cyanotic, and a thrombus may be palpable within the renal vein [34]. In the post-operative setting, transplant RVT manifests with sudden onset of oliguria/anuria, haematuria and severe pain/tenderness over the graft area [60]. The clinical presentation can help distinguish between different post-transplant vascular complications, since renal artery thrombosis has absent or minimal abdominal pain [60].

Finally, RVT can also be an incidental finding detected on abdominal imaging, especially in patients with cancer or NS [10,18]. The proportion of asymptomatic patients ranged from 7–15% [14,22] to 66–87% [10,18] in different studies, based on the characteristics of the enrolled population. Indeed, differences were noted between cancer and non-cancer patients. For instance, acute RVT was more frequently observed in non-cancer patients, whereas chronic and asymptomatic RVT presentations were more common in cancer patients (in non-cancer patients 85% were acute, 5% chronic, 10% asymptomatic; in cancer patients 42.1% were acute, 36.8% chronic, 21.1% asymptomatic; *p* = 0.01) [14].

Other symptoms can arise from extension of a left RVT into the tributaries of the left renal vein. Thrombosis of the left adrenal vein can lead to concomitant ipsilateral adrenal haemorrhage (reported in 8–32% of cases) [11,15,16]. Thrombosis of the left gonadal vein can lead to pelvic congestion in females or varicocele and testicular oedema in males [21,24,61].

## 5. Diagnosis

Renal venography used to be the gold standard imaging for the diagnosis of RVT. However, it is rarely used nowadays, because it is an invasive procedure that involves radiation and a contrast agent, and carries risks of venous injury and clot dislodgment [28].

Doppler ultrasonography (US) is a non-invasive imaging test which can be performed at the bedside, thus being the most common diagnostic test used in neonates and infants with suspicion of RVT. However, it is operator-dependent, and certain conditions (such as obesity and ascites) might impair the visualisation of the renal veins [28]. Older studies reported a sensitivity of 85–100% and a specificity of 56–75%, when compared to venography [62,63]. Doppler US can demonstrate the thrombus within the renal vein while evaluating renal structure and perfusion, with severely reduced perfusion serving as a poor prognostic indicator in RVT [64]. In general, in the early stages of RVT, the kidney is enlarged with echogenic intermedullary stripes (representing thrombosis of the small interlobar and interlobular veins), the renal vein shows absence of flow, and the renal artery shows reversed end-diastolic flow together with raised resistive indices [65]. After a few days, collateral circulation starts forming and the flow within the renal artery normalises. After 2–3 weeks, calcifications appear in the renal parenchymal (corresponding to calcified thrombi within the small veins), and ultimately the kidney may develop scarring and atrophy [64,66].

Nowadays, CT venography is the main imaging technique used to diagnose RVT. Advantages include the direct visualisation of the thrombus within the renal vein and other abdominal vessels, thus determining the extension of the thrombosis. Disadvantages include the use of ionising radiation and nephrotoxic iodinated contrast agent, which may pose a risk in patients with severe kidney impairment. A study conducted in patients with NS reported that CT has a sensitivity of 92.3% and specificity of 100% compared to renal venography [28].

**Figure 2:**
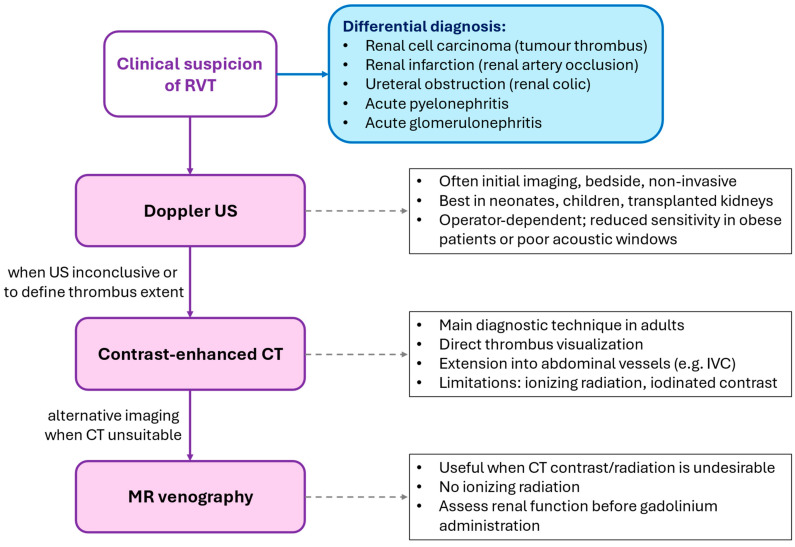
Diagnostic algorithm for renal vein thrombosis. Legend: CT = computed tomography, IVC = inferior vena cava, MR = magnetic resonance, RVT = renal vein thrombosis, US = ultrasound.

Magnetic resonance imaging (MRI) venography can also be used as an alternative to CT venography. Indeed, MRI does not use ionising radiation, and the gadolinium contrast agent does not cause AKI, though Group 1 (linear) gadolinium-based contrast agents may potentially lead to nephrogenic systemic fibrosis particularly if estimated glomerular filtration rate (GFR) <15 mL/min/1.73 m^2^ [67]. In a study including patients with NS, three-dimensional contrast-enhanced MRI venography was associated with a sensitivity of 94.1% and a specificity 100% compared to CT venography [68]. The diagnostic algorithm for RVT is summarised in Figure 2.

RVT should be distinguished from tumour thrombosis of the renal vein, i.e., spreading of a renal tumour into the renal vein. At contrast-enhanced CT, bland thrombus shows homogeneous enhancement, while tumour thrombus shows inhomogeneous enhancement [69]. At contrast-enhanced MRI, bland thrombus shows no obvious enhancement, while tumour thrombus shows enhancement [70]. Positron emission tomography (PET)-CT can also help differentiate active tumour thrombus from bland thrombus [71]. In patients with renal cell carcinoma, bland and tumour thrombus can coexist, a situation which complicates surgery and carries worse prognosis than tumour thrombosis alone [72]. Finally, while the use of artificial intelligence could aid in the early detection of thrombosis, its performance so far has been assessed mainly in patients with DVT and/or PE [73], while unusual thrombosis locations (such as RVT) have not yet been evaluated.

## 6. Treatment

Early reports on RVT were published between the late 1920s and early 1960s. At that time, urgent nephrectomy was considered the standard of care, and RVT-related mortality was high [3]. From the 1970s onwards, observational studies progressively included cases of RVT treated with medical therapy [3]. Nowadays, anticoagulation is the main treatment for bland RVT, traditionally low molecular weight heparin (LMWH) or unfractionated heparin (UFH) followed by vitamin K antagonists (VKA), although recent evidence emerged on the use of the direct oral anticoagulants (DOACs). Isolated tumour thrombosis of the renal vein does not typically respond to anticoagulation and might require oncological management.

### 6.1. Adult Cohort Studies

The most recent cohort studies of RVT in adult patients, mainly with an observational retrospective design, are summarised in Table 1. In the study by Mohamed et al., all 182 patients with RVT received anticoagulation (initially parenteral heparin, followed by VKA) [19]. In the study by Wysokinski et al., 114 out of 218 patients with RVT (52.3%) received heparin and 74 (33.9%) received warfarin. Warfarin treatment emerged as a predictor of survival (hazard ratio [HR] for mortality 0.53, 95% CI 0.31–0.90) [22]. However, this cohort included approximately 28% of patients with tumour thrombosis. Unfortunately, separate outcome data for bland RVT and tumour thrombus were not reported, which limits the interpretation of the role of anticoagulation [22]. In the study by Rottenstreich et al., 28 out of 39 patients with RVT (71.8%) were anticoagulated, and in this cohort there was a high proportion of thrombosis resolution (30/32 patients, 93.8%) [14]. In the study by Wanaratwichit et al., 40 out of 87 patients with RVT (46.0%) received long-term anticoagulation: LMWH (*n* = 17), warfarin (*n* = 22), and DOAC (*n* = 1). There were four major bleeding (10%) and six overall bleeding (15.0%) cases during anticoagulant treatment. Recanalization occurred in 14/40 (35.0%) patients receiving anticoagulation and in 8/47 (17.0%) patients who did not receive anticoagulation. Anticoagulant therapy had no effect on progression of kidney dysfunction (HR 1.32, 95% CI 0.51–3.44) or overall mortality (HR 0.91, 95% CI 0.54–1.50) [18].

Anticoagulant treatment duration in the above-mentioned studies varied. In Wysokinski et al., 34 out of 74 patients receiving warfarin (45.9%) were prescribed long-term treatment [22]. In Wanaratwichit et al., the median duration of anticoagulation was 52 days [18]. In Rottenstreich et al., anticoagulation was administered for 3–12 months in patients with RVT provoked by certain risk factors (e.g., surgery), while an indefinite duration was prescribed to patients with persistent risk factors (e.g., persistent NS) [14]. In the study by Mohamed et al., anticoagulant duration ranged from 1 year to life-long, particularly in patients with persistent risk factors or recurrent RVT [19]. However, the available studies did not provide detailed guidance on treatment duration. Therefore, lacking specific recommendations, general anticoagulation principles [74] may be applied, such as a minimum treatment duration of 3–6 months; limited duration for patients with transient risk factors (e.g., surgery, cancer in remission, or NS currently in remission); and extended duration for patients with unprovoked VTE or persistent risk factors (e.g., high-risk thrombophilia, active cancer or active NS with persistent nephrotic state).

Tumour thrombus in the renal vein requires a different approach, mainly surgical resection and/or chemotherapy. Although tumour thrombus may increase the risk of developing VTE, it is still uncertain whether prophylactic anticoagulation provides a net clinical benefit, as it appears to prevent only approximately half of VTE while increasing the risk of major bleeding [75].

**Table 1:** Cohort studies of renal vein thrombosis in adult patients.

Author (Year)	N. Patients with RVT	RVT Site (%)	IVC Thrombosis (%)	Age	Males (%)	Main Risk Factors (%)	Treatment (*n*)	Follow-Up Time	Key Results
**Wysokinski (2008)** [22]	218 (62 with tumour thrombus)	Left 43% Right 33% Bilateral 21%	43%	55.2 years (mean)	65%	Malignancy (66%) Nephrotic syndrome (20%)	Heparin (*n* = 114) VKA (*n* = 74) Aspirin (*n* = 22) IVC filter (*n* = 5) Thrombolysis (*n* = 1)	42.3 months (mean)	• Mortality: 138 patients (18.0/100 patient-years) • Recurrent VTE: 8 patients (1.0/100 patient-years) • Major bleeds: 4/218 (2%)
**Ross (2017)** [13]	17	Left 59% Right 6% Bilateral 35%	65%	46.3 years (mean)	59%	Nephrotic syndrome (100%)	VKA (*n* = 17) Thrombectomy (*n* = 2)	49 months (mean)	• Recurrent VTE: 0 • Mortality: 1/17 (6%)
**Rottenstreich (2017)** [14]	39	Left 44% Right 41% Bilateral 15%	46%	58 years (median)	54%	Malignancy (49%) Nephrotic syndrome (21%) Infection (13%)	Anticoagulation (*n* = 28) Nephrectomy with thrombectomy (*n* = 6) No specific treatment (*n* = 5)	35 months (mean)	• Thrombus resolution: 30/32 (94%) • Kidney atrophy: 2/32 (6%) • Recurrent VTE: 5/32 (16%) • Mortality: 9/32 (28%)
**Zhang (2018)** [76]	10 (out of 16 VTE)	Left 60% Right 30% Bilateral 10%	NR	21–35 years (median, whole cohort)	81% (of the whole cohort)	Nephrotic syndrome (100%)	Rivaroxaban (*n* = 4) LMWH (*n* = 6)	4 weeks	• Thrombus dissolution: 7/8 (88%) in rivaroxaban group, 7/8 (88%) in LMWH group • Major bleeds: 0 • Rivaroxaban similar efficacy and safety as LWMH
**Shinkawa (2021)** [17]	11 (out of 221 VTE)	NR	NR	39 years (median)	45%	Nephrotic syndrome (100%)	Any anticoagulant (*n* = 11) Heparin (*n* = 6) Antiplatelet (*n* = 3)	51 days (median)	• Mortality: 0
**Ahmed** **(2023)** [77]	8	Left 75% Right 13% Bilateral 13%	50%	64 years (median)	63%	Malignancy (75%) Nephrotic syndrome and oral contraceptive (13%) Infection (13%)	Rivaroxaban (*n* = 6) Apixaban (*n* = 2)	34 months (median)	• Recanalization: 7 patients (88%) • Clinically significant bleeding during anticoagulation: 2 patients (25%) • Recurrent VTE: 1 patient (13%)
**Oktaviana (2023)** [6]	10 (out of 113 abdominal VTE)	Left 50% Right 50%	20%	64.2 years (mean)	60%	Malignancy (50%) Infection (30%)	Enoxaparin (*n* = 1) VKA (*n* = 3) DOAC (*n* = 2) No specific treatment (*n* = 4)	37.0 months (mean)	• Recurrent VTE: 0 • Major bleeds: 0 • Mortality: 3/10 (30%)
**Zhang (2023)** [21]	40	Left 45% Right 25% Bilateral 30%	NR	37 years (median)	63%	Nephrotic syndrome (75%) Malignancy (13%)	Anticoagulation (*n* = 40) Thrombolysis or thrombectomy (*n* = 23)	20 months (median)	• Acute phase (<14 days): endovascular treatment higher thrombus clearance, better renal function • Subacute phase (14-30 days): endovascular treatment higher thrombus clearance, no difference in renal function • PE: 8/40 (20%)
**Wanaratwichit (2024)** [18]	87	Left 68% Right 24% Bilateral 8%	18%	57 years (median)	44%	Malignancy (61%) Post-surgery or trauma (16%) Nephrotic syndrome (13%)	LMWH (*n* = 17) VKA (*n* = 22) DOAC (*n* = 1) No specific treatment (*n* = 47)	1129 months (total time)	• Mortality: 57/87 (66%) • Worsening kidney function: 18/84 (21%) • Bleeding during anticoagulation: 6/40 (15%) • Recanalization: 22/33 (67%)
**Mohamed (2025)** [19]	182	Unilateral 80% Bilateral 20%	NR	47.1 years (mean)	42%	Nephrotic syndrome (52%) Malignancy (33%) Post-trauma or surgery (11%)	UFH and VKA (*n* = 182) Thrombolysis (*n* = 34) Thrombectomy (*n* = 64)	≥1 year	• Mortality: 82/182 (45%) • Fatal PE: 4/182 (4%) • Worsening kidney function: 126/182 (69%) • Acute RVT (<14 days): endovascular treatment better renal function, higher thrombus clearance • Chronic RVT (>14 days): endovascular treatment higher thrombus clearance, no difference in renal function

Note: Some of these cohorts included a small number of paediatric patients despite a predominantly adult population. Legend: DOAC = direct oral anticoagulants, IVC = inferior vena cava, LMWH = low molecular weight heparin, NR = not reported, PE = pulmonary embolism, RVT = renal vein thrombosis, UFH = unfractionated heparin, VKA = vitamin K antagonists, VTE = venous thromboembolism.

### 6.2. Paediatric Cohort Studies

Recent cohort studies of RVT in paediatric patients are summarised in Table 2. In the study by Marks et al., only 28 out of 43 neonates with RVT received specific treatments: 17 (39.5%) LMWH, 7 (16.3%) UFH, 1 (2.3%) VKA, and 3 (7.0%) thrombolysis [23]. In the study by Kosch et al. among 59 neonates with RVT, 33 (55.9%) were anticoagulated with either LMWH or UFH, 11 (18.6%) underwent thrombolysis, and 11 (18.6%) received supportive therapy only. However, a high proportion of patients developed renal atrophy during follow-up (81.8% of patients treated with thrombolysis, 90.9% of those receiving anticoagulation, and 90.9% of those not receiving any antithrombotic therapy) [11]. In the study by Ndoudi Likoho et al., among 27 neonates with RVT, 19 (70.4%) received LMWH, 2 (7.4%) UFH, 4 (14.8%) underwent thrombolysis, while 2 (7.4%) received no specific treatment. All patients treated with thrombolysis had bilateral RVT; the other four patients with bilateral RVT received either LMWH (*n* = 3) or supportive treatment only (*n* = 1). The use of thrombolysis in bilateral RVT was associated with higher rates of bleeding events compared to LMWH/supportive treatment (75% vs. 0%, *p* = 0.05) and similar rates of thrombus resolution (75% vs. 75%, *p* = 1.00), although these findings should be interpreted with caution due to the small number of patients [12].

A case-series of 72 neonatal RVT was reported from the “1-800-NO-CLOTS”, a Canadian-based telephone consultation service for clinicians dealing with paediatric VTE [7]. Treatment recommendations were based on whether the RVT was unilateral or bilateral (with or without extension to the IVC), and whether there was associated renal insufficiency. No treatment was recommended only for 29% of patients with unilateral RVT without renal insufficiency. Overall, most patients were given either UFH or LMWH, while VKA was suggested only for a few patients with bilateral RVT (8–14%). Thrombolysis was considered mainly in patients with renal insufficiency (57–100% of cases) [7].

Anticoagulant treatment duration in paediatric cohorts was generally shorter than in adult cohorts. In the study by Zigman et al., UFH was administered for 6 to 14 days, and LMWH for 14 days to 3 months [15]. Indeed, the median duration of UFH therapy was 18.5 days in the study by Bokenkamp et al. [9], while Bidadi et al. reported a median treatment duration of 6 months in a cohort primarily treated with LMWH [20]. In the study by Ndoudi Likoho et al., anticoagulant treatment with UFH or LMWH was administered for 4 to 12 weeks (median duration 8 weeks), with no significant differences between unilateral and bilateral RVT (*p* = 0.29). There were no significant differences in clot resolution between patients treated less than 6 weeks and those treated more than 6 weeks (partial resolution 71.4% vs. 73.4%, complete resolution 28.6% vs. 13.3%, respectively, *p* = 0.46) [12]. Only few paediatric patients received long-term VKA [2]. There are no specific recommendations about anticoagulant treatment or re-imaging in paediatric RVT. General guidelines for paediatric VTE suggests an anticoagulant treatment duration of 6 weeks for selected patients with provoked VTE (mainly due to resolved transient risk factors), as long as there is no persistent occlusive thrombus, and 6–12 months for unprovoked VTE [78].

**Table 2:** Cohort studies of renal vein thrombosis in paediatric patients.

Author (Year)	N. Patients with RVT	RVT Site (%)	IVC Thrombosis (%)	Age	Males (%)	Main Risk Factors (%)	Treatment (*n*)	Follow-Up Time	Key Results
**Bökenkamp (2000)** [9]	35	Unilateral 80% Bilateral 20%	29%	3 days (median)	NR	Prematurity (43%)	UFH (*n* = 23) Thrombolysis (*n* = 4) No specific treatment (*n* = 8)	11.5 months (median)	• Kidney atrophy: 26/39 affected kidneys (67%)
**Zigman (2000)** [15]	23	Left 48% Right 30% Bilateral 22%	52%	13 days (mean)	52%	Respiratory distress (30%) Foetal distress (26%) Maternal diabetes (17%)	UFH or LMWH (*n* = 12) VKA (*n* = 1) No specific treatment (*n* = 11)	46 months (mean)	• Renal function impairment: 7/7 (100%) in no-anticoagulation group, 3/9 (33%) in anticoagulation group • Anticoagulation improved renal outcome
**Kosch (2004)** [11]	59 (case–control study) 94 (follow-up study)	Left 39% Right 34% Bilateral 27%	25%	Neonates	59%	Sepsis (17%) Central venous line (15%) Birth asphyxia (12%)	LMWH (*n* = 28) UFH (*n* = 5) Thrombolysis (*n* = 11) AT concentrates (*n* = 4) No specific treatment (*n* = 11)	4.0 years (median)	• Kidney atrophy: 53/59 (90%) • Recurrent VTE: 4/94 (4%)
**Marks (2005)** [23]	43	Unilateral 56% Bilateral 44%	72%	Neonates	65%	Umbilical venous catheter (16%) Perinatal asphyxia (12%)	LMWH (*n* = 17) UFH (*n* = 7) VKA (*n* = 1) Thrombolysis (*n* = 3)	3.7 years (median)	• Mortality: 3/43 (7%) • VTE recurrence: 0 • Renal failure: 11/38 (29%) • Kidney atrophy: 25/38 (66%)
**Messinger (2006)** [16]	28	Left 54% Right 40% Bilateral 11%	46%	Neonates	71%	Central vascular line (32%) Foetal distress (25%) Maternal diabetes (18%)	UFH or LMWH (*n* = 10) Thrombolysis (*n* = 7)	4.3 years (median)	• Mortality: 2/28 (7%) • Kidney atrophy: 13/28 (46%) • Thrombolysis may prevent renal failure in bilateral RVT
**Winyard (2006)** [2]	23	Left 30% Right 13% Bilateral 57%	50%	1 day (median)	74%	Foetal distress (61%) Emergency caesarean section (48%) Intrauterine growth retardation (22%)	UFH (*n* = 2) LMWH (*n* = 2) VKA (*n* = 1) PC concentrates (*n* = 1)	64 months (mean)	• Mortality: 1/23 (4%) • Impaired renal growth or structure: 28/34 kidneys (82%) • Longer kidney length at presentation correlated with poorer renal outcome
**Bidadi (2016)** [20]	10	Left 50% Right 10% Bilateral 40%	50%	2 days (median)	90%	Prematurity (60%) Perinatal asphyxia (60%) Maternal diabetes (30%)	UFH or LMWH (*n* = 6) Thrombolysis (*n* = 2) AT concentrates (*n* = 2) No specific treatment (*n* = 4)	44 months (median)	• Recurrent VTE: 0 • Kidney atrophy: 8/10 (80%) • Anticoagulation or thrombolysis did not prevent renal atrophy • Thrombolysis may prevent worsening of renal failure in bilateral RVT
**Ouellette (2020)** [4]	85	NR	NR	1 day (median)	64%	Respiratory distress syndrome (52%) Congenital heart disease (29%) Maternal preeclampsia (20%) Maternal diabetes (20%)	NR	14.8 years (median)	• Composite outcome (CKD, all-cause mortality, hypertension): 49/85 (58%) • Combined outcome (CKD, all-cause mortality): 39/85 (46%)
**Ndoudi-Likoho (2023)** [12]	27	Left 41% Right 30% Bilateral 30%	74%	2.5 days (median)	59%	Prematurity (44%) Perinatal asphyxia (41%) Maternal diabetes (22%)	LMWH (*n* = 19) UFH (*n* = 2) Thrombolysis (*n* = 4) No specific treatment (*n* = 2)	5.7 years (median)	• Mortality: 1/27 (4%) • Recurrent VTE: 0 • Kidney atrophy: 18/26 (69%) • In bilateral RVT, fibrinolysis more bleeding than LMWH, no difference in clot resolution
**Whitworth (2023)** [79]	11 (out of 40 VTE)	Unilateral 64% Bilateral 36%	64%	Infants	M>F	Unprovoked (100%)	UFH or LMWH (*n* = 6) No specific treatment (*n* = 5)	7.8 years (median)	• Recurrent VTE: 1/11 (9%) • Kidney atrophy: 5/10 (50%)

Legend: AT = antithrombin, CKD = chronic kidney disease, IVC = inferior vena cava, LMWH = low molecular weight heparin, NR = not reported, PC = protein C, RVT = renal vein thrombosis, UFH = unfractionated heparin, VKA = vitamin K antagonists, VTE = venous thromboembolism.

### 6.3. Role of Direct Oral Anticoagulants

Since patients with unusual-site VTE were not included in the main trials evaluating the DOACs, there are only a few cases reported in the literature of patients with RVT treated with these drugs (Table 1 and Table 3) [45,48,49,50,53,77,80,81,82,83,84,85,86,87,88,89,90]. In addition, concerns have been raised regarding the use of DOACs in patients with NS and reduced kidney function, given their predominant renal clearance (ranging from 27% for apixaban to 80% for dabigatran). Hypoalbuminemia may also affect the DOAC pharmacokinetics by increasing their unbound fraction, which can lead to increased anticoagulant effect but also increased clearance, with high variability in drug exposure. Evidence from two retrospective studies including 21 and 27 patients with NS, respectively, suggests that thromboprophylaxis with DOACs (apixaban or rivaroxaban) is associated with a low incidence of thrombotic events, without an increased risk of bleeding [91,92].

A small pilot study conducted in China randomised 16 patients with VTE associated with NS and low antithrombin levels to receive dalteparin 5000 U twice daily or rivaroxaban 30 mg/day for 4 weeks; among these, 10 patients had RVT (6 in the LMWH group and 4 in the rivaroxaban group) [76]. All patients were evaluated at 2 and 4 weeks with radiological imaging, to assess the primary endpoint of thrombosis dissolution or reduction (>90% decrease in thrombus volume). Rivaroxaban showed identical rates of the primary endpoint (7 out of 8 patients in each group, 87.5%, at 4 weeks’ follow-up) and no major bleeding events [76]. However, these results should be interpreted with caution given the small number of patients with RVT. Two ongoing prospective studies are evaluating apixaban profile when administered to patients with NS (NCT04278729, NCT04850378). Due to lack of robust randomised controlled trials, the DOACs are not currently recommended for VTE prophylaxis or treatment in patients with NS and their use for this indication remains off-label [93]. Anti-Xa monitoring may be considered in these situations, although the therapeutic plasma concentration range has not been clearly established.

Ahmed et al. described a case-series of eight patients with RVT of various aetiology treated with rivaroxaban or apixaban and reported favourable outcomes: seven patients (87.5%) had partial or complete recanalization, two patients (25.0%) clinically relevant bleeding on treatment (defined as bleeding associated with any medical intervention), and one patient (12.5%) recurrent VTE after anticoagulant discontinuation [77]. Another 8 patients with RVT were included in the DUST study, an international registry of 349 patients with unusual-site VTE treated with DOACs with the aim of evaluating the rationale for choosing a DOAC) [94].

Lacking specific evidence on the use of DOACs in RVT, caution should be applied. While DOACs may be considered without concerns regarding efficacy in selected RVT patients, their use is not recommended in certain underlying conditions commonly associated with RVT, such as NS (due to unpredictable drug exposure [93]) and luminal genitourinary cancer (due to the higher bleeding risk [26]).

### 6.4. Endovascular Procedures

Endovascular procedures (mechanical thrombectomy, local catheter-directed thrombolysis, or ultrasound-mechanical thrombolysis) are usually reserved for more severe cases, such as bilateral acute RVT causing kidney dysfunction. Kim et al. reported six patients with acute RVT (defined as duration of symptoms <14 days), both on native and allograft renal veins, treated with percutaneous catheter-directed thrombectomy or thrombolysis, followed by UFH and VKA. All patients achieved venous recanalization and clinical improvement, as evidenced by decreased creatinine levels and increased GFR after a mean of 6.7 days [95].

Another two retrospective observational studies suggested that the benefit is observed only in acute RVT. In the study by Zhang et al., endovascular treatment was performed in cases of impaired renal function or no improvement in thrombus volume after 5 days of standard anticoagulation [21]. Out of 40 patients enrolled, 17 received anticoagulation alone, while 23 were treated with anticoagulation and endovascular procedures. Among patients with RVT in the acute phase (i.e., <14 days from symptoms onset), endovascular treatment was associated with higher thrombus clearance (100% vs. 63%, *p* = 0.049) and better renal function (mean GFR after treatment 128 vs. 110 mL/min/1.73 m^2^, *p* = 0.037; serum creatinine 53 vs. 75 µmol/L, *p* = 0.033) after treatment, compared to anticoagulation alone. Among patients with RVT in the subacute phase (i.e., 14–30 days from symptoms onset), endovascular treatment was associated only with higher thrombus clearance (91% vs. 33%, *p* = 0.017), but no difference in renal function (GFR 106 vs. 104 mL/min/1.73 m^2^, *p* = 0.91; creatinine 68 vs. 74 µmol/L, *p* = 0.401). After discharge, all patients received warfarin as long-term anticoagulation, which was associated with further thrombus clearance in 13 out of 19 patients with residual thrombosis (68%) [21].

In the study by Mohamed et al., endovascular treatment was performed in cases of anticoagulation failure, bilateral RVT, extension to IVC or acute kidney dysfunction. Out of 182 patients enrolled, 84 received anticoagulation alone and 89 were treated with anticoagulation and endovascular procedures. In patients with acute RVT (<14 days), the endovascular group showed better kidney function (mean serum creatinine after treatment 1.58 vs. 2.41 mg/dL, *p* = 0.047) and higher thrombus clearance (61.3% vs. 38.7%, *p* = 0.004). In patients with chronic RVT (>14 days), the endovascular group showed higher thrombus clearance (57.5% vs. 42.3%, *p* < 0.001), but no difference in kidney function (creatine 2.29 vs. 2.92 mg/dL, *p* = ns) [19]. In a recent systematic review and meta-analysis, thrombolytic therapy in paediatric VTE was associated with an increase, although not statistically significant, in the risk of major bleeding, clinically relevant non major bleeding, and unspecified bleeding [96]. However, most of the included studies involved patients with DVT and/or PE, while the evidence specifically addressing RVT was very limited (only two small case-series, for a total of seven patients with RVT treated with thrombolytic therapy) [96].

Recent case reports have described the use of ultrasound-facilitated catheter-directed thrombolysis in patients with extensive RVT [97,98,99]. Ultrasound waves may enhance the penetration of thrombolytic drugs into the fibrin clot, potentially allowing for lower drug doses, faster treatment and fewer complications. However, results to date have been mixed. Successful recanalization was obtained in some cases [97,98], while no substantial reduction in thrombus was achieved in another [99].

### 6.5. Guidelines

There are currently no specific guidelines for the treatment of RVT in adult patients, while the latest recommendations for paediatric cases are provided in the 2024 American Society of Hematology (ASH)/International Society on Thrombosis and Haemostasis (ISTH) guidelines [78]. In general, the guidelines suggest anticoagulation for neonatal RVT (recommendation 9) and distinguish two scenarios based on RVT prognosis: if RVT is life-threatening (e.g., bilateral RVT), they suggest thrombolysis followed by anticoagulation (recommendation 10b); if RVT is non-life-threatening (e.g., unilateral RVT with or without IVC extension), they recommend anticoagulation alone (recommendation 10a) [78]. However, these recommendations are based on very low certainty on the benefit of anticoagulation/thrombolysis in neonatal RVT, as the available literature consists mainly of small observational studies with a high risk of bias, and their benefit on long-term outcomes remains uncertain.

## 7. Prognosis

RVT can be associated with IVC thrombosis in up to two-thirds of patients [12,13,23,79]. Thrombosis extension has also been described retrogradely into the lower extremities [79] or progressively to the right atrium [14,22]. RVT may be a source of embolism and lead to concomitant PE, which has been reported in 12–18% of cases [13,14]. Left RVT can also extend into tributaries of the left renal vein (e.g., left adrenal vein and left gonadal vein) [11,24].

In Wysokinski et al., the rate of recurrent VTE was 1.0 per 100 patient-years during a mean follow-up of 42.3 months. The proportion of patients with RVT developing recurrent VTE was significantly lower than the proportion of patients with lower limb DVT (8/218, 3.7%, vs. 50/287, 17.4%, respectively, *p* < 0.001) [22]. Mortality rate in patients with RVT was 18.0 per 100 patient-years and was higher than patients with DVT and age- and sex-matched residents in the United States. Malignancy (HR 2.4, 95% CI 1.2–4.7) and infection (HR 2.4, 95% CI 1.4–4.0) were significant predictors of mortality, while warfarin treatment (HR 0.53, 95% CI 0.31–0.90) was a predictor of survival. Of note, in this study, 28.4% of patients had tumour thrombus [22]. In the study by Wanaratwichit et al., mortality rate was 52.1 per 100 patient-years in Thailand. Malignancy (HR 5.45, 95% CI 2.58–11.54), hypoalbuminemia (HR 2.88, 95% CI 1.65–5.05) and advanced age ≥ 75 years (HR 3.44, 95% CI 1.49–7.93) were significant predictors of mortality [18]. In the study by Mohamed et al., 82 out of 182 patients with RVT (45.1%) in Egypt died during a follow-up of at least one year. Malignancy (HR 6.15, 95% CI 1.79–21.09), bilateral RVT (HR 5.61, 95% CI 2.82–11.16) and diabetes mellitus (HR 2.42, 95% CI 1.28–4.59) were associated with increased mortality risk [19].

In paediatric cohorts, mortality was lower (4–7% of cases) [2,12,16,23], and more frequent complications included deterioration of kidney function and development of kidney atrophy. In a cohort of 11 infants with unprovoked RVT, two thirds developed abnormal kidney function (estimated GFR < 90 mL/min/1.73 m^2^) during a median follow-up of 7.8 years, and 90% showed kidney abnormalities at US imaging [79]. In a study of 23 perinatal RVT, more than 80% of cases had abnormal kidney imaging (reduced kidney growth or structural abnormalities) during a mean follow-up of 64 months. Kidney size at the time of RVT presentation negatively correlated with the outcomes: for every 1 mm increase in perinatal kidney length there was a GFR reduction of ~3 mL/min/1.73 m^2^ and an increase of 1.11 times in the odds of abnormal kidney imaging [2]. In different studies, renal atrophy ranged from 50% to 80% of paediatric cases [20,79].

In the study by Mohamed et al., 72 out of 182 adult patients with RVT (39.6%) developed AKI and 54 (29.7%) developed chronic kidney disease. Diabetes mellitus (odds ratio [OR] 14.04, 95% CI 3.38–58.37) and NS (OR 6.41, 95% CI 1.81–22.71) were associated with increased risk of renal dysfunction [19]. In the study by Wanaratwichit et al., the incidence rate of worsening kidney function was 19.1 per 100 patient-years. The presence of NS (HR 18.41, 95% CI 1.57–216.04), malignancy (HR 9.10, 95% CI 1.05–78.63) and body weight ≥ 60 kg (HR 4.82, 95% CI 1.43–16.32) were associated with increased risk of worsening kidney function, while symptomatic acute RVT onset (HR 0.12, 95% CI 0.01–0.96) was protective [18].

Another possible long-term complication of paediatric RVT is the development of hypertension, with varied rates reported in different studies: 2 out of 27 neonates (7.4%) followed up for a median of 5.7 years in Ndoudi Likoho et al. [12]; and 13 out of 40 neonates (32.5%) followed up for a median of 3.7 years in Marks et al. [23]. In Kosch et al., 13 out of 59 neonates with RVT (22.0%) developed severe arterial hypertension during a median follow-up of 4.0 years and required long-term anti-hypertensive medications; one case also required a nephrectomy. The authors noted that all patients with hypertension had renal atrophy (unilateral in 10 cases and bilateral in 3 cases) [11]. In Ouellette et al., 33 out of 85 neonates with RVT (38.8%) developed long-term hypertension after a median time of 11.1 years, compared to only 2.1% of healthy neonates without RVT in Canada. The corresponding incidence rates were 5.32 per 100 person-years in neonatal RVT vs. 0.18 in the comparator group. After adjusting for sex and comorbidities, the risk of developing hypertension in neonates with RVT was 15.7-fold higher than in healthy neonates without RVT [4].

## 8. Prevention

### 8.1. Renal Vein Thrombosis Prevention in Kidney Transplantation

Anticoagulant therapy is integrated into several stages during renal transplantation to minimise the incidence of allograft thrombosis. It is common practice to administer heparin (300 IU/kg) to the donor following declaration of brain death or withdrawal of life-sustaining treatment after circulatory death prior to aortic cross-clamping [100,101,102,103]. In the rare event that a donor presents with heparin-induced thrombocytopenia (HIT) or HIT with thrombosis (HITT), heparin may be substituted with argatroban, which may be administered as a 200 mg infusion over 15 min before aortic cross-clamping [104,105]. A similar approach may also be considered when the recipient has a history of HIT/HITT. Following retrieval, the graft is first flushed on the backbench with cold heparinised perfusion solution to evacuate residual blood and prevent micro-thrombi formation. Intra-operatively, the recipient may receive a systemic dose of heparin (e.g., 30–60 IU/kg) before vessel clamping at the surgeon’s discretion. In recipients with HIT/HITT, this may be replaced with danaparoid boluses when indicated. Finally, to ensure the patency of the vascular anastomoses, the recipient iliac arteriotomy and venotomy sites are irrigated with cold heparinised saline to clear air and micro-clots [106]. To further mitigate the risk of VTE in the post-operative period, prophylaxis is maintained through low-dose LMWH (or danaparoid in HIT/HITT) alongside mechanical interventions, such as thromboembolic deterrent stockings and intermittent pneumatic compression, tailored to the recipient’s specific bleeding and thrombotic risk profile [107].

### 8.2. Renal Vein Thrombosis Prevention in Nephrotic Syndrome

#### 8.2.1. Risk Assessment

Albeit a significant risk factor for thromboembolic events, there remains some controversy regarding the criteria for initiating prophylactic anticoagulation and the agent of choice in the presence of NS. Several studies have consistently demonstrated that the risk of VTE increases incrementally with the severity of hypoalbuminaemia, rather than the degree of proteinuria alone [108], with Anti-Phospholipase-A2 Receptor Antibody-positive primary MN amongst the glomerulopathies carrying the highest thrombotic risk [109,110]. The first six months following a diagnosis of NS are also associated with the highest incidence of VTE episodes [111].

Prophylactic anticoagulation is recommended when the risk of venous thromboembolism exceeds the risk of bleeding. The Kidney Disease: Improving Global Outcomes (KDIGO) guidelines suggest prophylactic anticoagulation in adults with severe hypoalbuminaemia (serum albumin <20–25g/L) in the presence of any of the following risk factors: proteinuria >10 g/day, body mass index >35 kg/m^2^, genetic predisposition for thromboembolism, prolonged periods of immobilisation, recent major high-risk surgery, or moderate-to-severe symptomatic heart failure [93]. The 2021 KDIGO algorithm is based on data from patients with primary MN, thus its applicability to individuals with other causes of NS may be challenging. The UK Kidney Association (UKKA) guidelines mirror the 2021 KDIGO recommendations [112], and include central venous access as an additional risk factor for thrombosis in children and young adults with NS [113].

Lee et al. constructed an additional tool for use in individuals with primary MN, enabling estimation of bleeding risk in relation to the potential benefits of anticoagulation, based on the acceptable benefit-to-risk ratio for the patient and clinician [114]. This risk assessment tool takes into account additional comorbidities based on the Anticoagulation and Risk Factors in Atrial Fibrillation (ATRIA) bleeding risk score (although not validated for use in NS), highlighting the importance of a personalised approach when considering anticoagulation. This tool has not been validated in patients with NS beyond primary MN, and its applicability to other causes of NS therefore remains undetermined, although it serves as a good aide memoire during clinical risk assessment.

#### 8.2.2. Prophylactic Anticoagulation

KDIGO continues to recommend warfarin (target International Normalised Ratio 2-3) or LMWH (low- or full-dose) for VTE prophylaxis in both adults and children with NS [93], owing to the extensive long-term experience with their use, although they have not been compared directly. However, real-world data from the CureGN cohort indicate that DOAC use has become prevalent in clinical practice among adults [115]. Evidence supporting DOACs remains limited to retrospective studies and case series, suggesting potential benefit for VTE prophylaxis but with ongoing uncertainty due to altered pharmacokinetics in NS, including the risk of both over- and underexposure. Encouragingly, early pharmacokinetic data, particularly for apixaban, suggest acceptable safety and efficacy for VTE prophylaxis in NS [92,116,117] with further studies underway (NCT04278729, NCT04850378) [118]. Retrospective comparative studies between warfarin and DOACs showed similar bleeding and thrombotic episodes in patients with underlying NS [119,120]. Current UKKA paediatric guidelines do not exclude DOACs and recommend that anticoagulant choice be guided by local practice [113]. One may consider monitoring such cases with DOAC-specific anti-factor Xa levels to ensure satisfactory exposure to the drug.

If the bleeding risk is too high with full-dose anticoagulation, or there is a significant arterial thromboembolic (i.e., cardiovascular) risk, aspirin may also be considered as an alternative to anticoagulation [93,121]. However, the role of aspirin in VTE prevention remains unclear, with Lionaki et al. reporting that nearly half of patients with primary MN developed VTE whilst receiving antiplatelet therapy [108].

The optimal duration of anticoagulation therapy remains uncertain. The 2021 KDIGO guidelines [93] recommend continuing prophylaxis for as long as the NS persists, with decisions regarding ongoing low-dose prophylactic enoxaparin individualised to the patient. In the absence of timely randomised controlled trials, target trial emulation using large registry data represents an important approach to bridging evidence gaps in both DOAC use and optimal prophylaxis duration.

## 9. Conclusions and Future Directions

RVT represents one of the least common sites of VTE. The literature shows significant variability in clinical presentation, management and outcomes, due to the rarity and the heterogeneous nature of RVT in adults and paediatric cases. Anticoagulation is the cornerstone of care, despite the evidence being derived from small cohort studies, mainly with a retrospective design. Given the risk of bleeding, thrombolysis is reserved for cases involving bilateral RVT or significant renal impairment. There is a lack of studies specifically assessing the DOACs in RVT and of specific guidelines for the adult population. There is also a lack of studies exploring the quality of life (QoL) of patients with RVT and their adherence to anticoagulant treatment. The burden of long-term anticoagulant treatment, the risk of recurrent thrombosis, and the presence of renal impairment are all factors which may affect patient-reported outcomes. While validated QoL questionnaires are available for patients with lower-extremity DVT or PE (e.g., VEINES-QOL/Sym [122] or PEmb-QoL [123]), chronic kidney disease (e.g., KDQOL-36 [124]), and anticoagulant therapy (e.g., PACT-Q [125] or ACTS [126]), there is no RVT-specific QoL questionnaire.

Future research should focus on large-scale, collaborative, and prospective studies incorporating standardised outcome measures [127] across both adult and paediatric populations. This will allow clarification of optimal management strategies, refinement of prognostic indicators, and address gaps in evidence regarding long-term outcomes and the safety and efficacy of the anticoagulant treatment. Future research should also consider qualitative studies examining how RVT affects patients’ QoL, including their physical, psychological, and social wellbeing, while also exploring both patient experiences and professional perspectives on care. Such efforts will be critical in advancing care for patients with RVT while minimising uncertainty where the literature remains inconclusive.

## Figures and Tables

**Table 3:** Case reports of patients with renal vein thrombosis treated with the direct oral anticoagulants.

Author (Year)	Age	Sex	Site RVT	Risk Factors	Anticoagulant Therapy Details	Outcomes
**Dupree (2014)** [80]	18	Female	Left renal vein	Nephrotic syndrome	UFH → Warfarin (3 months) → Rivaroxaban 15 mg BID for 21 days, then 20 mg OD (6 months)	• PE on warfarin • No recurrent VTE on DOAC
**Shimada (2017)** [81]	39	Male	Right renal vein + PE	Nephrotic syndrome	UFH → Warfarin (1 month) → Edoxaban 30 mg OD (8 months)	• New PE on warfarin • No recurrent VTE on DOAC • RVT and PE resolution
**Date (2019)** [82]	64	Male	Left renal vein	Post-surgery for abdominal aortic aneurysm	Apixaban 5 mg BID (6 months)	• No recurrent VTE • RVT resolution
**Matta (2019)** [83]	44	Female	Right renal vein + PE	Unprovoked	UFH → Rivaroxaban 15 mg BID for 21 days, then 20 mg OD (1 year)	• Clinical improvement • No recurrent VTE
**Asleson (2022)** [50]	44	Female	Left renal vein	COVID-19 infection	Enoxaparin → Rivaroxaban 15 mg BID for 21 days, then 20 mg OD (3 months)	• RVT resolution • No recurrent VTE
**Hsu (2022)** [84]	41	Female	Right renal vein + IVC	Hormonal therapy for infertility + COVID-19 vaccine	Thrombolysis + Rivaroxaban 15 mg OD (5 months)	• RVT resolution • No recurrent VTE
**Zhu (2022)** [85]	11	Male	Bilateral renal veins + IVC + PE	Mycoplasma pneumoniae infection + AT deficiency	Thrombolysis + Rivaroxaban 10 mg BID for 1 day, then 5 BID for 2 days, then 10 BID (3 months)	• New PE, new IVC thrombosis, new right RVT, and iliac veins thrombosis on rivaroxaban • No further recurrent VTE after increasing DOAC dose
**Kakhktsyan (2023)** [49]	39	Male	Left renal vein	COVID-19 infection	Apixaban 5 mg BID (6 months)	• RVT resolution • No recurrent VTE
**Miyahara (2023)** [86]	36	Female	Left renal vein	Oral contraceptives + smoking	UFH → Edoxaban (dose not reported) (6 months)	• RVT resolution • No recurrent VTE
**Watanabe (2023)** [45]	69	Male	Bilateral renal veins + PE	Autoimmune haemolytic anaemia + eosinophilic granulomatosis polyangiitis	UFH → Apixaban (dose not reported) (6 months)	• RVT and PE resolution • No recurrent VTE
**Chawla (2024)** [53]	29	Female	Right renal vein	Oral contraceptives + obesity	LMWH → Apixaban (dose not reported) (3 months)	• Clinical improvement
**Low (2024)** [87]	21	Female	Left renal vein	Hormonal contraceptives (intra-vaginal ring)	Thrombectomy + thrombolysis → UFH → Apixaban 5 mg BID (6 months)	• RVT resolution • No recurrent VTE
**Nascimento (2024)** [88]	67	Male	Right renal vein (at the confluence with IVC)	Blunt renal trauma with renal haematoma	LMWH + IVC filter placement → Rivaroxaban 10 mg OD (3 months) → IVC filter removal + Rivaroxaban 15 BID (1 month)	• RVT resolution
**De Masi De Luca (2025)** [89]	34	Male	Left renal vein + PE	Nephrotic syndrome	Fondaparinux → Rivaroxaban 15 mg BID for 21 days, then 20 mg OD (3-4 months)	• RVT and PE resolution
**Hegele (2025)** [48]	21	Female	Left renal vein	Urinary tract infection + oral contraceptives + vaping nicotine and cannabis use	LMWH → UFH → Apixaban 5 mg BID (3 months)	• RVT resolution • No recurrent VTE
**Moorani (2025)** [90]	10	Female	Right renal vein + IVC	Antiphospholipid syndrome + systemic lupus erythematosus	LMWH → Warfarin → Rivaroxaban 10 mg OD, later reduced to 5 mg OD due to menorrhagia (4 years)	• Persistent RVT on warfarin • No recurrent VTE

Legend: AT = antithrombin, BID = twice daily, COVID = coronavirus disease, DOAC = direct oral anticoagulant, IVC = inferior vena cava, OD = once daily, PE = pulmonary embolism, RVT = renal vein thrombosis, UFH = unfractionated heparin, VTE = venous thromboembolism.

## Data Availability

No new data were created or analysed in this study. Data sharing is not applicable to this article.
